# How drawing practice enhances distress tolerance in university students: the mediating roles of psychological resilience and self-disclosure

**DOI:** 10.3389/fpsyg.2025.1540900

**Published:** 2025-07-09

**Authors:** Ziwei Lyu, Shuangping Ouyang, Shuxin Zhang

**Affiliations:** ^1^College of Art and Design, Hunan First Normal University, Changsha, China; ^2^School of Mechanical Engineering and Mechanics, Xiangtan University, Xiangtan, China; ^3^International College, National Institute of Development Administration, Bangkok, Thailand

**Keywords:** drawing practice, psychological resilience, self-disclosure, distress tolerance, university students

## Abstract

**Introduction:**

University students often face significant academic and emotional pressures, making distress tolerance a vital skill for maintaining mental wellbeing. Drawing practice, as a creative outlet, has been shown to promote emotional regulation and psychological health. This study examines how drawing practice influences distress tolerance, focusing on the mediating roles of psychological resilience and self-disclosure.

**Methods:**

This study recruited 354 university students from Hunan Province, China, using a combination of convenience sampling and snowball sampling methods to ensure a diverse and representative participant pool. These approaches facilitated efficient data collection while capturing varied student experiences. To test the proposed hypotheses, a structural equation model (SEM) was developed and analyzed using AMOS, allowing for a robust evaluation of the relationships among the variables.

**Results:**

This study examined the relationship between drawing practice and distress tolerance, focusing on the mediating roles of psychological resilience and self-disclosure. The results indicated that drawing practice is associated with higher psychological resilience and greater self-disclosure, both of which are linked to improved distress tolerance. These findings underscore the indirect pathways connecting drawing practice to distress tolerance.

**Discussion:**

This study highlights how drawing practice contributes to university students' ability to manage academic and emotional pressures by fostering psychological resilience and encouraging self-disclosure, both of which are linked to better distress tolerance. These findings demonstrate the potential of creative activities like drawing to support university students' mental wellbeing, providing valuable insights for promoting emotional health in high-stress academic environments.

## 1 Introduction

University students often face significant academic and emotional pressures, making mental health a pressing issue for this population (Valdes et al., 2022). According to a 2023 study by Renmin University of China, the total number of students in China is ~293 million, with a mental health problem detection rate of 18.9%. Among these, internalizing problems (such as anxiety, depression, sleep issues, and suicidal thoughts) account for 20.0%, while externalizing problems (such as self-harm and suicide attempts) have a detection rate of 11.7%. Specifically, among 1,333,593 university students, the mental health problem detection rates, in descending order, are as follows: sleep problems (23.5%), depression (20.8%), self-harm (16.2%), anxiety (13.7%), suicidal thoughts (10.8%), somatization (4.5%), and suicide attempts (2.7%). These figures clearly indicate the immense academic pressures and emotional struggles that university students face, highlighting the growing prominence of mental health issues (Li et al., 2021). Emotional regulation plays a crucial role in maintaining mental health (Albagawi et al., 2024). Distress tolerance refers to an individual's ability to withstand and cope with emotional distress (Landi et al., 2023; Leonelli et al., 2023). For university students, enhancing distress tolerance is not only helpful in managing academic stress and emotional challenges but also in reducing the occurrence of mental health issues. Therefore, exploring ways to improve distress tolerance among university students is of particular importance. By adopting effective emotional regulation strategies, students can better manage stress, promote mental health, and ultimately improve their overall quality of life.

In current emotional management approaches, although various interventions such as psychological counseling (Agirkan, 2023), cognitive behavioral therapy (Hofmann and Smits, 2008), and medication have been proposed, they face limitations in terms of accessibility, cost, and potential side effects (Cadel et al., 2021; Halbesleben et al., 2013). First, many existing emotional management strategies require significant financial resources and time commitments, which may be unrealistic for university students with limited economic means. For instance, psychological counseling often involves regular fees and a complex appointment process, which can prevent students from receiving timely support (Sanberk and Akbas, 2015). Additionally, traditional mental health interventions tend to focus on verbal expression and cognitive restructuring, which, while effective to some extent, overlook the potential of creative activities in emotional regulation (Mitschke et al., 2017; Pham et al., 2021).

Against this backdrop, drawing practice has emerged as a potential intervention gaining attention (Casati, 2021). Drawing is intuitive, flexible, and low-barrier, making it not only a cost-effective option that can be widely promoted, but also easily integrated into daily life, catering to a diverse range of audiences (Ågren and Aarsand, 2024). More importantly, existing research suggests that drawing provides a safe outlet for emotional expression and stimulates creativity and imagination, enabling individuals to express their inner feelings and pressures in a non-verbal manner (Rellensmann et al., 2017; Scholtes, 2023). For example, studies have shown that individuals engaged in drawing activities are better able to find emotional outlets when faced with distress, facilitating positive emotional expression and release (Kastner et al., 2021). Specifically, the literature supports the positive effects of drawing practice for children with autism spectrum disorder (Drake, 2023). Some studies have found that drawing can significantly reduce symptoms of anxiety and depression (Cheung et al., 2018), with participants in drawing activities reporting higher self-expression abilities and emotional relief (Chen et al., 2013). These findings indicate that drawing is not just an artistic activity, but also an effective tool for emotional regulation. Although preliminary evidence has supported the psychological benefits of drawing, there remains a lack of systematic quantitative research exploring how drawing practice influences distress tolerance through underlying mechanisms. In particular, few studies have examined the potential mediating roles of psychological resilience and self-disclosure in this relationship. This study aims to fill this gap by constructing and empirically testing a model that explains how drawing practice, as a creative activity, can enhance students' capacity for distress tolerance via these two psychological pathways.

Psychological resilience refers to an individual's capacity to recover from adversity and adapt positively to stress, making it a crucial factor in managing emotional challenges (Mishra et al., 2024). Resilient individuals are more likely to adopt effective coping strategies, maintain a strong sense of purpose, and respond to stressful environments with greater emotional stability (Prayag et al., 2020). For university students, developing resilience is especially important, as it helps buffer the negative effects of academic and social pressures, thereby supporting their mental wellbeing in challenging circumstances (Mao et al., 2023). At the same time, self-disclosure plays a vital role in psychological health and in building supportive social networks (Kumano, 2002). It involves the intentional sharing of one's personal thoughts, emotions, and experiences. Research has shown that moderate levels of self-disclosure can foster intimacy in interpersonal relationships and facilitate the receipt of social support, both of which contribute to improved psychological functioning (Piko et al., 2022). For university students, the ability to openly express emotional concerns to others may help reduce feelings of loneliness and anxiety while enhancing their sense of psychological safety.

Drawing practice, with its inherently reflective and expressive nature, offers students a safe, non-verbal medium through which they can externalize complex emotions and experiences (Burkitt and Lowry, 2015; You, 2023). By engaging in this form of artistic expression, students are able to develop greater self-awareness and improve their emotional regulation skills. This process may also promote self-disclosure, as individuals who express their emotions through art may become more comfortable articulating those same emotions to others. In addition, drawing activities support emotional release and introspection, allowing students to explore internal struggles and challenges in a constructive manner (Uchinokura, 2020). The repetitive and creative aspects of drawing can also help build resilience by encouraging perseverance, enhancing problem-solving abilities, and fostering a sense of accomplishment (Munro, 2016). Thus, these mechanisms suggest that drawing practice, as an intervention, has the potential to enhance distress tolerance in university students by increasing psychological resilience and self-disclosure.

This study holds both theoretical and practical significance in advancing the understanding of creative activities as tools for psychological intervention. Theoretically, it seeks to uncover the specific mechanisms through which drawing practice influences distress tolerance, addressing a critical gap in the current literature on non-traditional mental health interventions. By investigating the mediating roles of psychological resilience and self-disclosure, this research contributes to a deeper understanding of how creative activities can foster emotional regulation and mental wellbeing, thereby complementing and expanding existing approaches in psychological intervention studies. Practically, the findings of this study provide valuable insights for designing and implementing mental health support programs in higher education institutions. As universities grapple with increasing rates of mental health challenges among students, this research offers an evidence-based rationale for integrating low-cost, accessible creative activities like drawing into campus mental health initiatives. Such programs could serve as effective and inclusive strategies to enhance students' distress tolerance, emotional resilience, and overall psychological health.

The structure of this paper is organized as follows: Section 2 outlines the research hypotheses and conceptual models. Section 3 provides a detailed description of the methods employed for data collection and analysis. Section 4 presents the results of the data analysis and tests the proposed hypotheses. Section 5 offers a discussion that includes theoretical contributions, practical implications, limitations, and suggestions for future research. Finally, Section 6 concludes the paper.

## 2 Literature review and hypothesis development

### 2.1 Social cognitive theory

Social Cognitive Theory (SCT), as developed by Bandura, provides a comprehensive framework for understanding how individuals regulate behavior and emotions through the dynamic interaction of cognitive, behavioral, and environmental influences (Bandura, 2001). SCT emphasizes the role of self-regulation, self-efficacy, and social modeling in shaping adaptive psychological responses to stress and adversity. Within this theoretical perspective, personal agency and observational learning are critical for the development of emotional and behavioral competencies, particularly in coping with emotional distress.

Previous literature has applied SCT to explain how individuals acquire and strengthen coping strategies through reflective and experiential learning processes (Zimmerman, 2000). Emotional regulation, for instance, is considered both a learned and self-directed behavior, often influenced by one's belief in their ability to manage internal states effectively (Bandura et al., 2003). Within the framework of SCT, drawing practice can be viewed as a self-reflective activity that enables individuals to process emotions, visualize internal experiences, and exercise self-directed coping behaviors. Through repeated engagement in drawing, individuals may strengthen psychological capacities such as resilience, which is considered a self-regulatory trait influenced by previous success in emotional coping and reinforced by perceived efficacy. At the same time, self-disclosure may be understood as a socially mediated behavior that emerges from internal emotional clarity and confidence in interpersonal contexts—two factors that SCT links to observational learning and self-appraisal.

Taken together, SCT supports a theoretical model in which drawing practice facilitates the development of internal resources (such as resilience) and interpersonal behaviors (such as self-disclosure), which in turn enhance the individual's distress tolerance, understood as the ability to endure emotional discomfort without maladaptive responses. The theory thus provides a coherent basis for examining the indirect pathways through which creative practices contribute to psychological adjustment.

### 2.2 Drawing practice, psychological resilience, and self-disclosure

Drawing practice, as a creative activity, has been explored in recent psychological research for its potential benefits in emotional expression, stress reduction, and psychological wellbeing (Ågren and Aarsand, 2024; Botma and Labuschagne, 2019). Creative outlets like drawing allow individuals to externalize internal emotions, facilitating emotional processing and offering a sense of control over otherwise overwhelming feelings (Burkitt and Lowry, 2015; Wigglesworth, 2017). Several studies have suggested that engaging in artistic activities can reduce anxiety, improve mood, and foster emotional resilience, making drawing an effective tool for psychological health, particularly among university students who face significant academic and social pressures (DeFauw, 2016; Poole and Dickinson, 2014).

Psychological resilience refers to an individual's ability to adapt and recover from adversity or stress (Prayag et al., 2020). It involves the development of coping mechanisms and emotional regulation strategies that help individuals maintain wellbeing despite challenges. Drawing, as a form of self-expression, can play a vital role in enhancing resilience (Rellensmann et al., 2017). Research has demonstrated that engaging in creative activities, including drawing, fosters psychological flexibility and helps individuals navigate difficult emotions by providing an outlet for expression (Graeme-Cook et al., 2024). Studies have also shown that creative practices can promote cognitive and emotional processing, which, in turn, can enhance an individual's ability to manage stress and recover from setbacks (Héroux, 2018). Drawing practice offers a way to externalize complex feelings, helping individuals to process these emotions in a structured, manageable way (Spendlove, 2007). It can also promote positive thinking and self-reflection, essential components of psychological resilience. Therefore, this study proposes the following hypotheses:

**Hypothesis 1 (H1):** *Drawing practice has a positive association with psychological resilience*.

Self-disclosure involves revealing personal thoughts, feelings, and experiences to others (Geller, 2003). It is considered a crucial factor in building strong social connections, fostering emotional support, and enhancing psychological wellbeing (Barnett, 2011). Self-disclosure helps individuals alleviate emotional distress by sharing their burdens with trusted others, which can contribute to reducing feelings of isolation and increasing social support (Piko et al., 2022; Constantine and Kwan, 2003).

Creative activities like drawing, particularly in a group setting or as part of therapeutic interventions, can facilitate self-disclosure by providing a non-verbal means of expressing emotions. Through the act of drawing, individuals may feel more comfortable externalizing their feelings, which can then lead to verbal self-disclosure in social or therapeutic contexts (Wilhelm and Zlotnick, 2014). Additionally, drawing may help individuals articulate emotions that are difficult to express with words, thus enhancing their ability to disclose personal thoughts and experiences in a meaningful way. This process may also help to build trust and open communication channels within social networks (Rains et al., 2016). Therefore, it is hypothesized that drawing practice not only enhances emotional expression but also encourages self-disclosure, particularly when it helps individuals organize and articulate their internal emotional experiences. Therefore, this study proposes the following hypotheses:

**Hypothesis 2 (H2):** *Drawing practice has a positive association with self-disclosure*.

The relationship between psychological resilience and self-disclosure is multifaceted and supported by existing literature, though differing perspectives exist. Some researchers suggest that psychological resilience influences self-disclosure. Individuals with higher resilience are better equipped to manage their emotions, which may make them more inclined to share their feelings (Mittler et al., 2014). Studies have shown that resilient individuals possess stronger emotional awareness and confidence in their ability to handle challenging emotions (Brown et al., 2021). This confidence reduces fear of negative evaluation or vulnerability, enabling them to disclose their emotions more freely. Conversely, other scholars argue that self-disclosure may enhance psychological resilience by fostering emotional support and building adaptive coping mechanisms (D'Agata et al., 2021). Sharing emotions and experiences with others can strengthen interpersonal bonds, which in turn helps individuals develop resilience (Liao et al., 2024).

In the context of drawing practice, this study posits that psychological resilience serves as a key factor in facilitating self-disclosure. Drawing provides a reflective and expressive outlet, enabling individuals to process and externalize complex emotions. As resilience grows through this creative engagement, it may encourage individuals to disclose their inner thoughts and feelings more openly, particularly within a safe and supportive environment. Therefore, based on this reasoning, the following hypothesis is proposed:

**Hypothesis 3 (H3):** *Psychological resilience has a positive association with self-disclosure*.

### 2.3 Psychological resilience, self-disclosure and distress tolerance

Distress tolerance refers to the ability to withstand and effectively manage negative emotional states, preventing them from escalating into maladaptive behaviors or emotional crises (Rogers et al., 2018). It is a critical psychological resource that helps individuals navigate adversity and stress without becoming overwhelmed. For university students, who often experience academic pressures, social challenges, and emotional turbulence, distress tolerance is crucial for maintaining mental wellbeing and achieving academic success (Veilleux et al., 2019). Although distress tolerance has been widely discussed in psychological research, the factors that contribute to its development are less clearly defined, particularly when it comes to the roles of psychological resilience and self-disclosure (Veilleux, 2023).

Psychological resilience involves the capacity to adapt to stress and adversity in a way that promotes recovery and wellbeing (Prayag et al., 2020). Resilient individuals are often better equipped to regulate their emotions and cope with negative situations in a healthy manner, which is likely to contribute to their ability to tolerate distress (Serçe et al., 2021). While the direct relationship between psychological resilience and distress tolerance has not been quantitatively examined in existing literature, theoretical models suggest that resilience enhances distress tolerance through the development of adaptive coping mechanisms, emotional regulation skills, and an overall greater ability to manage and recover from stress (Bacchi and Licinio, 2017).

Resilient individuals are typically more adept at handling challenging situations without becoming overwhelmed, suggesting that resilience might act as a buffer against the emotional strain that often leads to low distress tolerance (Gearhart et al., 2024). Furthermore, as resilience encourages emotional flexibility and adaptive thinking, it enables individuals to view stressful events as manageable challenges, rather than insurmountable threats (van Doorn and Hülsheger, 2015). This ability to frame stress in a less threatening way is crucial for sustaining distress tolerance in difficult situations. Given these theoretical underpinnings, it is hypothesized that:

**Hypothesis 4 (H4):** *Psychological resilience has a positive association with distress tolerance*.

Self-disclosure, defined as the act of revealing personal feelings, experiences, or thoughts to others, has long been recognized as a key factor in promoting psychological wellbeing (Piko et al., 2022). Previous studies have shown that self-disclosure can help individuals process and regulate their emotions, thereby contributing to greater emotional stability (Rains et al., 2016). While no direct quantitative studies have specifically explored the relationship between self-disclosure and distress tolerance, it can be logically inferred that the process of self-disclosure plays a role in enhancing distress tolerance. By sharing emotions and experiences, individuals may reduce emotional burden and gain perspective, leading to a greater sense of control over stressful situations (Bolton et al., 2003).

Self-disclosure also strengthens social support, which is crucial for coping with distress (Karsay et al., 2019). By expressing personal emotions and receiving feedback from trusted others, individuals can experience a sense of connection and validation, which can alleviate emotional strain and enhance their ability to tolerate distress (Yardeni et al., 2024). This social validation likely plays a key role in reducing feelings of isolation, which are often exacerbated under stressful conditions. Building on these theoretical insights, it is hypothesized that:

**Hypothesis 5 (H5):** *Self-disclosure has a positive association with distress tolerance*.

### 2.4 Mediation effects

While no previous research has directly examined the mediating effects of psychological resilience and self-disclosure in the context of drawing practice and distress tolerance, existing theoretical and empirical studies suggest that both variables are key mechanisms through which drawing practice can influence distress tolerance.

Drawing practice, as a form of creative expression, has been shown to facilitate emotional processing and regulation (Drake, 2023). Engaging in drawing provides individuals with a non-verbal outlet for expressing emotions, which can contribute to improved emotional awareness and regulation. This process is believed to promote psychological resilience by encouraging individuals to confront and process negative emotions in a constructive manner. Drawing has been linked to reduced anxiety, enhanced mood, and a greater sense of control over emotions, all of which are key components of psychological resilience (Casati, 2021).

Additionally, drawing practice can serve as a form of self-expression, which might encourage self-disclosure, either internally (e.g., reflecting on personal emotions and experiences) or externally (e.g., sharing one's artwork with others). Previous research has suggested that engaging in creative activities like drawing can foster communication and self-expression, which may lead to greater openness and self-disclosure (Héroux, 2018). Self-disclosure, in turn, is associated with the development of social support networks and improved emotional regulation, both of which are important for enhancing distress tolerance (Karsay et al., 2019).

Psychological resilience, as discussed earlier, helps individuals adapt to adversity and recover from stress (Bacchi and Licinio, 2017). By engaging in drawing practice, individuals may enhance their psychological resilience through improved emotional regulation and coping mechanisms. Resilient individuals are more likely to approach distressing situations with a problem-solving mindset, which enables them to endure and manage stress without becoming overwhelmed (van Doorn and Hülsheger, 2015). Therefore, it is plausible to suggest that drawing practice, by fostering psychological resilience, can indirectly improve distress tolerance. Self-disclosure also plays a critical role in managing emotional distress. By sharing emotions and experiences with others, individuals can reduce emotional burdens, receive emotional validation, and gain a greater sense of control over their distress (Guo et al., 2019). As drawing practice may encourage self-expression and the sharing of emotions, it is reasonable to infer that self-disclosure could serve as a mediator between drawing practice and distress tolerance. By allowing individuals to process and express emotions, self-disclosure may help them better manage stressful situations and improve their capacity to tolerate distress. Therefore, it is reasonable to hypothesize that both psychological resilience and self-disclosure mediate the relationship between drawing practice and distress tolerance. Thus, this study proposes the following hypothesis:

**Hypothesis 6 (H6):** *Psychological resilience and self-disclosure mediate the relationship between drawing practice and distress tolerance*.

**Figure 1:**
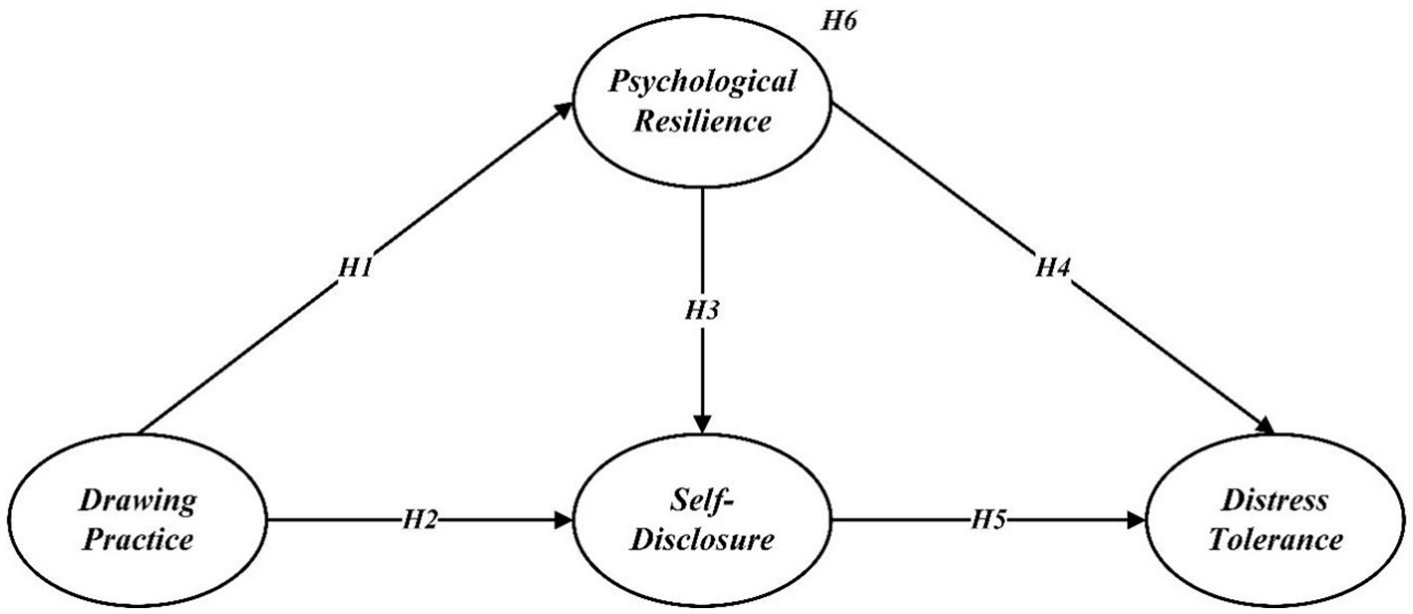
Hypothesis model.

All the hypotheses are outlined in Figure 1.

## 3 Methodology

### 3.1 Participants and procedures

The subjects of this study were university students from Hunan Province, as drawing practice is believed to play a significant role in fostering their psychological resilience and self-disclosure, which are critical during their developmental stage. It is also hypothesized that drawing practice can help them better cope with academic pressure and improve their emotional regulation and concentration. Researchers conducted an online questionnaire survey among university students from two universities in Hunan Province, China, in November 2024. The questionnaire was designed to assess various aspects of their participation in drawing practice and its impact on psychological and emotional factors. Data were collected using convenience sampling and snowball sampling methods, which allowed for the efficient gathering of a diverse and representative sample within a short timeframe. During the survey, all participants were informed of the purpose of the study and voluntarily completed the questionnaire to ensure the authenticity of the data and adherence to ethical standards. The survey was paused after collecting 400 responses. After excluding invalid questionnaires, a total of 354 valid responses were obtained, resulting in an effective response rate of 88.5%.

As shown in Table 1, the demographic characteristics of the 354 participants indicate a slightly higher proportion of female students (54.8%) compared to male students (45.2%). The largest groups were freshmen (32.5%) and seniors (30.2%), with most participants (50.3%) ranking in the middle 50% academically. Notably, 43.5% reported having received drawing-related training, and 42.4% spent 3 h or more daily on non-academic activities, highlighting a diverse range of experiences among respondents.

**Table 1:** Demographic characteristics (*n* = 354).

**Profiles**	**Item**	***n* (%)**
Gender	Male	160 (45.2)
	Female	194 (54.8)
Grade	Freshman	115 (32.5)
	Sophomore	59 (16.7)
	Junior	73 (20.6)
	Senior	107 (30.2)
Academic performance ranking	Top 25%	116 (32.8)
	Middle 50%	178 (50.3)
	Bottom 25%	60 (16.9)
Whether the respondent had received drawing-related training	Yes	154 (43.5)
	No	200 (56.5)
Average daily time spent on non-academic activities	1 h or less	81 (22.0)
	1–2 h	61 (17.2)
	2–3 h	62 (17.5)
	3 h or more	150 (42.4)

### 3.2 Instruments

The questionnaire consisted of five sections. The first section collected demographic information, including gender, grade, academic performance ranking, whether the respondent had received drawing-related training, and the average daily time spent on non-academic activities.

The second section included three items to assess the respondent's participation in drawing practice over the past week, specifically: “In the past week, I frequently participated in drawing activities,” “In the past week, I was able to fully concentrate on drawing without being distracted,” and “In the past week, I actively wanted to engage in drawing activities.” The third section measured psychological resilience using six items from Smith, Dalen (Smith et al., 2008), with a sample question: “I can handle stressful events effectively.” The fourth section assessed self-disclosure based on six items adapted from Miller, Berg (Miller et al., 1983), such as: “I openly share details about my close relationships with others.” The fifth section evaluated distress tolerance using four items from Garner, Van Kirk (Garner et al., 2018), including: “My feelings of distress are so intense that they completely take over.”

All sections of the questionnaire employed a five-point Likert scale. For sections two, three, and four, the scale ranged from 1 (Strongly Disagree) to 5 (Strongly Agree). For the fifth section, the scale ranged from 1 (Strongly Agree) to 5 (Strongly Disagree).

### 3.3 Data analysis

This study employed AMOS v.23 to construct a structural equation model (SEM) to explore the mechanisms by which drawing practice enhances university students' psychological resilience and self-disclosure, ultimately improving their distress tolerance. The analysis followed a rigorous two-step process, beginning with the evaluation of the measurement model to establish reliability and validity, and then moving on to assess the structural model. Parameters were estimated using the maximum likelihood (ML) method, ensuring robust and reliable estimates. Key analyses included testing the model's reliability, validity, fit indices, path coefficients, and mediating effects, providing a comprehensive understanding of the relationships among the variables.

To address the potential influence of common method variance (CMV), which can arise from the self-reported nature of the data, the procedure recommended by Mossholder, Bennett (Mossholder et al., 1998) was implemented. Specifically, a comparison between two models was conducted by analyzing their chi-square values and degrees of freedom. The first model produced a chi-square value of 3,122.829 with 170 degrees of freedom, while the second yielded a chi-square value of 369.908 with 146 degrees of freedom. Both models had *p*-values below 0.001, indicating statistical significance. These results suggest that the model fits the data well and that CMV does not pose a significant threat to the validity of the findings in this study.

## 4 Results

### 4.1 Measurement model

The reliability and validity of the latent variables were assessed using a combination of SPSS and confirmatory factor analysis (CFA) in AMOS v.23. Specifically, internal consistency reliability (e.g., Cronbach's α) was calculated using SPSS, and the results demonstrated excellent internal consistency across all constructs, with Cronbach's α values exceeding the widely accepted benchmark of 0.9, as shown in Table 2. This high level of reliability aligns with the recommendations of Fornell and Larcker (1981). Furthermore, the average variance extracted (AVE) for each construct was above the threshold of 0.7, and the composite reliability (CR) values exceeded 0.9, indicating robust convergent validity. These metrics confirm that the constructs adequately capture the variance of their respective indicators.

**Table 2:** Reliability and validity.

**Items**	**Factor loadings**	**Cronbach's α**	**CR**	**AVE**
**Drawing practice**		0.948	0.948	0.859
**(DP)**				
DP1	0.936			
DP2	0.921			
DP3	0.924			
**Psychological**		0.933	0.933	0.700
**resilience (PR)**				
PR1	0.837			
PR2	0.819			
PR3	0.813			
PR4	0.889			
PR5	0.835			
PR6	0.824			
**Self-disclosure (SD)**		0.941	0.941	0.728
SD1	0.829			
SD2	0.845			
SD3	0.870			
SD4	0.910			
SD5	0.877			
SD6	0.781			
**Distress tolerance**		0.919	0.921	0.746
**(DT)**				
DT1	0.855			
DT2	0.891			
DT3	0.913			
DT4	0.790			

In addition, the factor loadings derived from principal component analysis ranged from 0.781 to 0.936 (Table 2), providing further evidence of the measurement model's construct validity. Discriminant validity was also established by demonstrating that the square root of the AVE for each construct was greater than the correlations between that construct and others, as detailed in Table 3. This finding ensures that the constructs are distinct and measure unique aspects of the conceptual framework.

**Table 3:** Pearson correlation.

**Construct**	**DP**	**PR**	**SD**	**DT**
**DP**	(0.927)			
**PR**	0.399^**^	(0.837)		
**SD**	0.411^**^	0.399^**^	(0.853)	
**DT**	0.349^**^	0.651^**^	0.541^**^	(0.864)

The square root of the AVE is in diagonals; off diagonals are a Person's corrections of contracts.

** *p* < 0.01.

Together, these results validate the measurement model, confirming that it meets the rigorous standards of reliability and validity required for structural equation modeling. This strong foundation enhances the credibility of the subsequent analyses and supports the robustness of the study's findings.

### 4.2 Structural model

**Figure 2:**
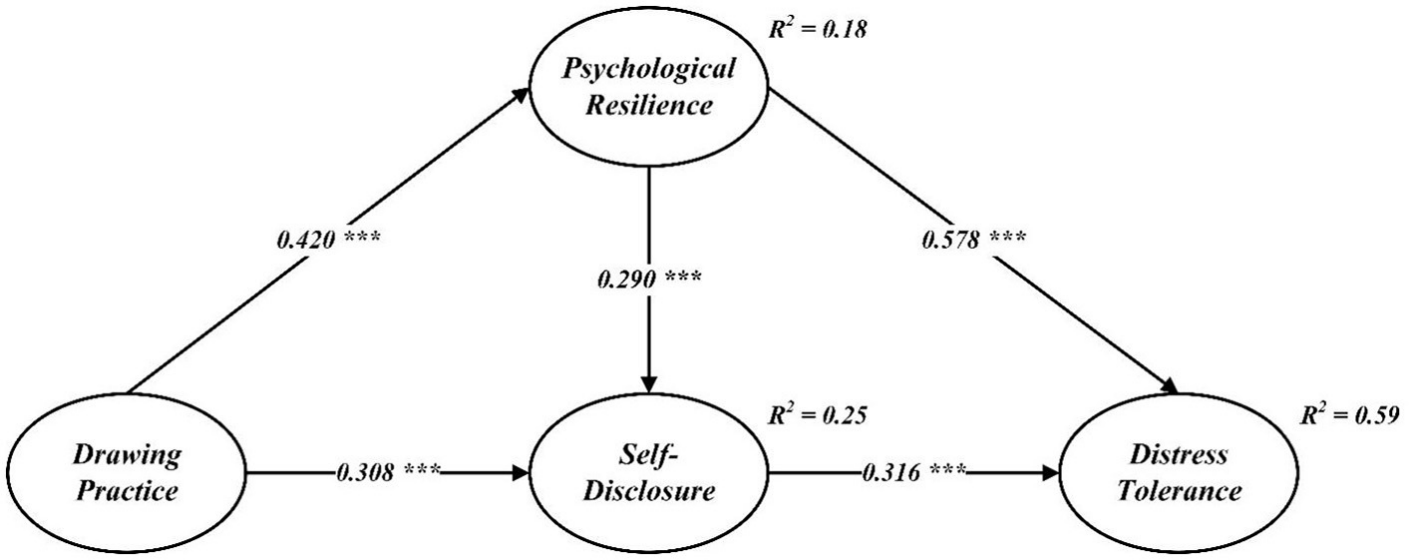
Structural path model. ^***^p < 0.001.

After confirming the reliability and validity of the measurement model, the structural model was evaluated using AMOS v.23 with 5,000 bootstrap samples to ensure robust results. The CFA demonstrated that the model fit the data well, with fit indices meeting or exceeding recommended thresholds (χ^2^/d*f* = 2.517, NFI = 0.941, IFI = 0.964, TLI = 0.958, CFI = 0.963). These metrics indicate that the hypothesized relationships and latent constructs align closely with the observed data, providing a solid foundation for hypothesis testing. Additionally, Table 3 reports the Pearson correlation coefficients, offering further evidence of the expected relationships among the study variables. Figure 2 visually represents the structural model, highlighting the standardized path coefficients.

As illustrated in Figure 2, drawing practice was positively and significantly associated with psychological resilience (β = 0.420, *p* < 0.001) and self-disclosure (β = 0.308, *p* < 0.001), supporting hypotheses H1 and H2. This finding underscores the role of drawing practice in fostering emotional and psychological strengths. Moreover, psychological resilience was positively linked to self-disclosure (β = 0.290, *p* < 0.001) and distress tolerance (β = 0.578, *p* < 0.001), affirming hypotheses H3 and H4. These results highlight the mediating role of resilience in enhancing both interpersonal openness and the capacity to endure emotional challenges. Finally, self-disclosure was found to have a positive relationship with distress tolerance (β = 0.316, *p* < 0.001), lending strong support to H5 and suggesting that open expression of thoughts and feelings can further strengthen an individual's ability to manage stress effectively.

The mediating effects were analyzed through a bootstrap procedure involving 5,000 resamples and 95% bias-corrected confidence intervals, as detailed in Table 4. The findings revealed that drawing practice significantly influenced distress tolerance indirectly through the dual mediating roles of psychological resilience and self-disclosure. This indirect effect was pronounced, with an estimated value of 0.378 (SE = 0.041, CI = [0.296, 0.458], *p* < 0.001), offering compelling evidence in support of hypothesis H6. These results underscore the pivotal role of psychological resilience and self-disclosure as mechanisms that link creative practices, such as drawing, to enhanced emotional endurance and adaptability.

**Table 4:** Standardized indirect effect.

**Standardized indirect effect**	**Point estimate**	**Product of coefficients**		**Bootstrapping**		
		**SE**	**Z**	**Bias-corrected 95% CI**		**Two-tailed significance**
				**Lower**	**Upper**	
DP → DT	0.378	0.041	9.220	0.296	0.458	*p* < 0.001

DP, drawing practice; DT, distress tolerance.

## 5 Discussion

### 5.1 Theoretical contributions

This study makes several significant theoretical contributions to the fields of psychological resilience, self-disclosure, and distress tolerance, particularly in the context of creative interventions such as drawing practice. By investigating the dual mediating roles of psychological resilience and self-disclosure in the relationship between drawing practice and distress tolerance, this research provides a novel and systematic framework for understanding how creative expression influences emotional wellbeing. Specifically, this study constructs a dual mediation model, which, for the first time, systematically tests the pathways linking drawing practice to distress tolerance through both psychological resilience and self-disclosure. This fills an important modeling gap by integrating artistic activity with emotional regulation mechanisms, thereby enhancing the theoretical systematization and novelty of the model.

First, this study contributes to the growing body of research on psychological resilience by demonstrating how creative activities like drawing can enhance resilience in the face of stress. Although previous studies have examined resilience as a predictor of emotional regulation and distress tolerance, the specific mechanisms through which resilience influences distress tolerance have not been thoroughly explored (Serçe et al., 2021). Our study bridges this gap by highlighting the role of drawing practice in fostering psychological resilience and, by extension, enhancing distress tolerance. This finding supports existing resilience theory, which posits that resilient individuals are better equipped to manage adversity, but it also expands our understanding by showing how an external activity like drawing can facilitate the development of resilience.

Second, this study advances the theory of self-disclosure by highlighting its mediating role between drawing practice and distress tolerance. While self-disclosure has been widely recognized as a key factor in emotional wellbeing, particularly in therapeutic contexts, the specific mechanisms through which it operates have not been fully explored (Liao et al., 2024). Our research extends the self-disclosure literature by demonstrating how engaging in drawing practice can encourage self-expression, which in turn enhances individuals' emotional regulation capabilities. This theoretical contribution is significant, as it links creative practices, typically viewed as individual or non-verbal activities, to interpersonal and emotional outcomes through the process of self-disclosure.

Furthermore, the study contributes to the understanding of distress tolerance, an essential but underexplored construct in the literature on emotional regulation. While distress tolerance has been identified as crucial for mental wellbeing, particularly in high-stress environments like academia, the factors that contribute to its development remain poorly understood. By investigating the role of psychological resilience and self-disclosure, this study provides a more nuanced theoretical framework for understanding how distress tolerance can be cultivated. It challenges the traditional view of distress tolerance as a static trait and offers a dynamic perspective in which creative activities, like drawing, can enhance an individual's capacity to tolerate distress over time.

Finally, this study contributes to the broader field of psychological interventions by providing a novel perspective on the potential of drawing practice as an accessible and effective tool for promoting mental health. While much of the research on creative activities focuses on their therapeutic benefits in clinical settings, our study demonstrates that such practices can have significant positive effects on non-clinical populations, such as university students. This theoretical insight has important implications for the design of mental health interventions, especially in high-pressure academic environments, and supports the notion that creative outlets can play a vital role in enhancing psychological resilience and emotional regulation.

In addition, this study extends Social Cognitive Theory by demonstrating how drawing practice functions as an interactive process involving personal cognitive factors (psychological resilience), behavioral engagement (drawing and self-disclosure), and environmental contexts (creative practice settings). By empirically validating the mediating roles of psychological resilience and self-disclosure, the findings reinforce SCT's concept of reciprocal determinism and self-regulation. This research highlights how individuals' engagement in creative behaviors can actively shape their cognitive and emotional capacities to tolerate distress, thereby expanding the application of SCT within the domain of psychological wellbeing and creative interventions.

In summary, this study provides valuable theoretical contributions by expanding the understanding of psychological resilience, self-disclosure, and distress tolerance in the context of creative activities. It offers a novel framework for how drawing practice can foster resilience and self-disclosure, ultimately improving distress tolerance, and provides a deeper theoretical foundation for future research in the area of emotional wellbeing and psychological interventions.

### 5.2 Practical implications

The findings of this study demonstrate significant relationships between drawing practice, self-disclosure, psychological resilience, and distress tolerance, offering valuable insights for practical applications across various high-stress populations. In particular, the study provides a foundation for incorporating drawing-based interventions into psychological support systems in an operationally feasible and cost-effective manner.

First, universities can integrate drawing practice into their counseling and peer-support programs as a low-cost intervention tool. For example, student counseling centers can offer guided drawing sessions facilitated by trained counselors or art therapy practitioners. These sessions may serve as part of routine mental health services, particularly during stressful periods such as exam seasons. Additionally, universities can introduce peer-led drawing clubs where students engage in expressive art together and share their reflections in a supportive environment. These activities not only encourage self-expression and self-disclosure but also foster a sense of community and emotional support.

Second, academic departments and student affairs offices can incorporate expressive arts modules into orientation programs or resilience-building workshops. For instance, a short-term “Creative Coping” program could combine guided drawing with group discussions, teaching students to use artistic expression as a tool for managing stress. Faculty members may also receive training to incorporate art-based reflective tasks—such as visual journals or emotion-mapping exercises—into their courses, encouraging emotional literacy and open dialogue in the classroom.

Third, these implications extend beyond university campuses. Adolescents, particularly those in high-pressure academic environments, may benefit from similar drawing-based interventions implemented through school counseling services or youth centers. Early-career professionals, who often face challenges such as work-related anxiety and role transitions, could participate in workplace wellness programs that include drawing activities for stress relief and self-reflection. Vulnerable populations, including individuals in community shelters, rehabilitation programs, or refugee settings, may also gain therapeutic benefits from structured drawing workshops aimed at enhancing emotional regulation and psychological resilience. These populations often lack access to conventional mental health care, and drawing serves as a low-threshold entry point for psychological support.

Fourth, families can play a supportive role by creating emotionally open home environments where drawing is encouraged as a means of communication and relaxation. Parents can promote drawing as a daily activity that fosters self-expression and resilience, particularly for adolescents dealing with emotional challenges. Simple practices like providing art supplies, initiating family art sessions, or encouraging children to illustrate their feelings can have long-term emotional benefits.

Finally, mental health professionals can integrate drawing practices into both individual and group therapy sessions across various age groups and settings. Guided drawing activities can help clients externalize and process difficult emotions, making them more receptive to further therapeutic work. Therapists can also teach clients structured self-disclosure techniques—such as drawing narrative timelines or emotion wheels—to bridge the gap between nonverbal expression and verbal reflection, thus improving overall distress tolerance.

In sum, this study offers a set of operationally applicable and broadly adaptable intervention strategies that underscore the value of creative practices in promoting mental health. By extending the applicability of drawing practice beyond university students to include adolescents, early-career professionals, and other vulnerable groups, this research highlights drawing as a flexible, accessible, and impactful tool for enhancing emotional wellbeing across diverse high-stress populations. Collaborative efforts among educators, policymakers, mental health practitioners, families, and individuals themselves are essential to translating these findings into practice.

### 5.3 Limitations

This study has several limitations. First, the variables examined were limited, and additional factors that might influence distress tolerance, such as social support or personality traits, were not included. Future research could incorporate a broader range of psychological and contextual variables to provide a more comprehensive understanding of the mechanisms underlying distress tolerance.

Second, the reliance on self-reported data may introduce biases, such as social desirability or recall inaccuracies, potentially affecting the validity of the findings. Subsequent studies are encouraged to combine self-report measures with behavioral assessments or third-party evaluations to enhance data reliability.

Finally, the use of convenience and snowball sampling may limit the generalizability of the results, as the sample may not fully represent the broader population of university students. Future research should consider employing stratified or random sampling techniques across diverse regions and institutions to improve the representativeness of the findings.

## 6 Conclusion

This study investigated the relationship between drawing practice and distress tolerance among 354 university students, with a focus on the mediating roles of psychological resilience and self-disclosure. The findings highlight the significant impact of drawing practice on both psychological resilience and self-disclosure, which, in turn, contribute to enhanced distress tolerance. This research not only underscores the potential of creative activities, such as drawing, as a practical and low-cost intervention for promoting emotional wellbeing in university students but also expands theoretical understanding by identifying key mediators in this process. The findings suggest that integrating drawing practice into university mental health programs could be an effective strategy for fostering resilience and emotional expression, ultimately improving students' ability to cope with academic and emotional pressures. Given the growing mental health challenges faced by university students, this research provides valuable guidance for developing accessible and impactful psychological support initiatives.

## Data Availability

The raw data supporting the conclusions of this article will be made available by the authors, without undue reservation.
